# Segmentation techniques of brain arteriovenous malformations for 3D visualization: a systematic review

**DOI:** 10.1007/s11547-022-01567-5

**Published:** 2022-10-18

**Authors:** Elisa Colombo, Tim Fick, Giuseppe Esposito, Menno Germans, Luca Regli, Tristan van Doormaal

**Affiliations:** 1 Department of Neurosurgery, Clinical Neuroscience Center and University of Zürich, University Hospital Zurich, Frauenklinikstrasse 10, 8091 Zürich, ZH Switzerland; 2 Prinses Màxima Center, Department of Neurosurgery, Utrecht, CS The Netherlands; 3 Department of Neurosurgery and Clinical Neuroscience Centerentrum, University Hospital of Zurich, Zürich, ZH Switzerland

**Keywords:** Cerebrovascular surgery, Cerebral arteriovenous malformation, Segmentation, Augmented reality, Blood vessel delineation

## Abstract

**Background:**

Visualization, analysis and characterization of the angioarchitecture of a brain arteriovenous malformation (bAVM) present crucial steps for understanding and management of these complex lesions. Three-dimensional (3D) segmentation and 3D visualization of bAVMs play hereby a significant role. We performed a systematic review regarding currently available 3D segmentation and visualization techniques for bAVMs.

**Methods:**

PubMed, Embase and Google Scholar were searched to identify studies reporting 3D segmentation techniques applied to bAVM characterization. Category of input scan, segmentation (automatic, semiautomatic, manual), time needed for segmentation and 3D visualization techniques were noted.

**Results:**

Thirty-three studies were included. Thirteen (39%) used MRI as baseline imaging modality, 9 used DSA (27%), and 7 used CT (21%). Segmentation through automatic algorithms was used in 20 (61%), semiautomatic segmentation in 6 (18%), and manual segmentation in 7 (21%) studies. Median automatic segmentation time was 10 min (IQR 33), semiautomatic 25 min (IQR 73). Manual segmentation time was reported in only one study, with the mean of 5–10 min. Thirty-two (97%) studies used screens to visualize the 3D segmentations outcomes and 1 (3%) study utilized a heads-up display (HUD). Integration with mixed reality was used in 4 studies (12%).

**Conclusions:**

A golden standard for 3D visualization of bAVMs does not exist. This review describes a tendency over time to base segmentation on algorithms trained with machine learning. Unsupervised fuzzy-based algorithms thereby stand out as potential preferred strategy. Continued efforts will be necessary to improve algorithms, integrate complete hemodynamic assessment and find innovative tools for tridimensional visualization.

## Introduction

Brain AVMs (bAVMs) are complex vascular lesions. Tailoring management for each patient is challenging and demands an accurate knowledge and understanding of the angioarchitecture of the malformation. bAVMs represent a relevant cause of secondary intracerebral hemorrhage, with a risk of rupture of approximately 1% yearly that on average increases fivefold after rupture [1–3].

Brain bAVMs are composed of feeding arteries and draining veins entangled in a nidus, without an intervening capillary bed [4]. Visualization of these structures is vital in the understanding of the angioarchitecture. Comprehension of flow direction and amount is necessary to grasp the hemodynamic effects. Determination of vessel positions in relationship to the nidus and brain structures is fundamental to optimize treatment strategies. The best imaging tools for the characterization of bAVMs are digital subtraction angiography (DSA) and magnetic resonance angiography (MRA)[5]. Both imaging modalities play a role in the diagnostic and perioperative management of these lesions, highlighting bAVMs’ features that drive their therapeutic management [6–8]. In neurosurgery, based on clinical and imaging findings, the severity of bAVMs, their predicted risk of rupture and necessity for intervention are mostly rated according to the classical Spetzler–Martin classification and its supplementation provided by Lawton et al. in 2010, demonstrating a stronger correlation with surgical outcomes [9–11].

Visualization of feeding arteries, nidus and venous drainage system is vital in the understanding of bAVM angioarchitecture and related hemodynamics: to tailor a therapeutic strategy, it is important to determine vessel positions and flow directions and to distinguish them depending on their nature and role. An accurate understanding of the angioarchitecture of bAVMs can be achieved by segmentation [3, 12–16]. Segmentation is intended as manual delineation of structures on a Digital Imaging and Communications in Medicine (DICOM) series to create its 3D shape. With segmentation, size and shape of cerebral structures can be measured to determine their spatial characterization and to plan a precise therapeutic intervention [16, 17]. Modern visualization techniques such as augmented reality (AR), 3D screens, as well as mixed reality (MR) and virtual reality (VR) have also been implemented to show segmentation results [18–22].

Several publications have reviewed vessel extraction techniques [15, 23–25]. They have helped to classify segmentation and visualization strategies and show the diversity and limitations of the used methods. Nonetheless, a major lack in the current literature is the presence of a gold standard technique shared by multiple centers worldwide to address segmentation of bAVMs, as well as the integration of hemodynamic information to characterize not only the morphology, but also the physiology of these complex lesions. The aim of this systematic review is to provide an up-to-date collection of on 3D methods to study the angioarchitecture of bAVMs for pretreatment planning and during embolization, surgery or radiosurgical treatment, as well as their integration to hemodynamic information and augmented reality rendering.

## Materials and methods

A systematic review was performed using the Preferred Reporting Items for Systematic Reviews and Meta-Analyses (PRISMA) guidelines [26]. Two reviewers (EC and TF) screened records independently, and disagreements at any stage were resolved by discussion and consensus. Two additional records were identified through reference search. Studies were excluded when considered beyond the scope for the aims of the present analysis, and/or when their outcomes were not of interest. The critical appraisal of the included studies was performed by means of risk of bias score as shown in Table 1.

**Table 1:** Descriptive analysis of the main data extracted from the 33 included studies

Author	Segmentation method	Imaging source	Treatment	Timing_min	Goal_vessels	Risk of bias A B C D
Muacevic A et al. [48]	Manual	CT	Microsurgery	NA	Feeders, nidus, veins	+ + + NA
Söderman M et al. [39]	Manual	DSA	Radiosurgery	NA	Volume	+—- NA
Coste E et al. [40]	Semiautomatic	DSA	Radiosurgery	NA	Volume	+—- NA
Bullitt E et al. [27]	Semiautomatic	MRI	Microsurgery	120	Feeders, nidus, veins	+ + + NA
Bullitt E et al. [58]	Semiautomatic	CT, MRI, DSA	Microsurgery	120	Nidus, nidus, veins	+—+ NA
Zhang XQ et al. [21]	Manual	CT, DSA	Radiosurgery	NA	Nidus	+—+ NA
Lee CC et al. (2003)	Semiautomatic	MRI	Radiosurgery	NA	Nidus	+—NA NA
Nyui Y et al. [49]	Automatic	CT	Radiosurgery	NA	Feeders, veins	+—+ NA
Coenen VA et al. (2005)	Semiautomatic	CT	Microsurgery	15	Feeders, nidus, veins	+ + + NA
Berger MO et al. (2008)	Semiautomatic	DSA	Radiosurgery	NA	Nidus	+—NA NA
Forkert ND et al. [28]	Automatic	MRI	Microsurgery	35	Feeders, nidus, veins	+ + + NA
Forkert ND et al. [29]	Automatic	MRI	Microsurgery	NA	Feeders, nidus, veins	+ + + NA
Forkert ND et al. [30]	Automatic	MRI	Microsurgery	NA	Feeders, nidus, veins	+ + + NA
Forkert ND et al. [31]	Automatic	MRI	Microsurgery	5	Feeders, veins	+—+ NA
Hristov D et al. [42]	Automatic	DSA	Radiosurgery	NA	Image source integration	+—+ NA
Babin D et al. [52]	Automatic	CT	Microsurgery	NA	Feeders, veins	+—+ NA
Babin D et al. [51]	Automatic	CT	Microsurgery	15	Feeders, nidus, veins	+ + + NA
Forkert ND et al. [32]	Automatic	MRI	Microsurgery	45	Image source integration	+—+ NA
Babin D et al. [25]	Automatic	CT	Microsurgery	2	Feeders, nidus, veins	+ + + NA
Forkert ND et al. [33]	Automatic	MRI	Microsurgery	5	Feeders, veins	+—+ NA
Clarencon et al. (2014)	Semiautomatic	DSA	Microsurgery	64	Feeders, nidus, veins	+ +—NA
Li F et al. (2014)	Automatic	DSA	Microsurgery	4 s per vessel	Feeders, nidus, veins	+ + + NA
Di Ieva et al. (2014)	Manual	MRI	Radiosurgery	NA	Feeders, nidus, veins	+ + + NA
Cabrilo I et al. (2014)	Automatic	CT, MRI, DSA	Microsurgery	NA	Feeders, veins	+—- NA
Li F et al. [44]	Automatic	DSA	Embolization	NA	Veins	+—+ NA
Phellan R et al. [35]	Manual	MRI	Microsurgery	NA	NA	+ NANANA
Peng SJ et al. (2018)	Automatic	MRI	Radiosurgery	NA	Nidus	+—+ NA
Babin D et al. [46]	Automatic	DSA	Embolization	NA	Veins	+—+ NA
Mascitelli JR et al. [55]	Manual	CT, MRI	Microsurgery	NA	NA	+ NANANA
Wang T et al. [54]	Semiautomatic	CT	Radiosurgery	NA	Volume	+—+ NA
Chenoune Y et al. [47]	Automatic	DSA	Embolization	NA	Feeders, nidus, veins	+ + + NA
Simon AB et al. [37]	Automatic	MRI	Radiosurgery	NA	Feeders, nidus, veins	+ + + NA
Mandel M et al. [39]	Manual	MRI	Microsurgery	10	Feeders, nidus, veins	+ +—NA

Risk of bias score legend

A: Appropriate eligibility criteria

B: Exposure/outcome measurement

C: Failure to adequately control confounding

D: Incomplete follow-up

*CT* computer tomography, *DSA* digital subtraction angiography, *MRI* magnetic resonance imaging, *NA* not applicable

### Search strategy

The PubMed, Embase and Google Scholar databases were searched to identify eligible papers. The query was performed using the Boolean operators “AND” or “OR” and database-related filters to maximize the chance to identify articles focusing on segmentation strategies and their 3D visualization specific of bAVMs. The string ((‘cerebral arteriovenous malformations’) AND (‘brain arteriovenous malformations’) AND segmentation OR ‘tridimensional visualization’ OR ‘3D’ OR ‘skeletonization methods’ OR ‘augmented reality’) was entered. The most recent search was performed on February 18, 2022.

### Selection criteria

Articles were included if all the following criteria were met. 1) Studies published after 1997; 2) studies analyzing specifically the angioarchitecture of brain arteriovenous malformations; 3) a 3D segmentation technique on top of source data as a mean to study angioarchitecture; 4) English, Italian, French or German language; 5) studies integrating the segmentation outcome with augmented reality technology.

### Data extraction

The following information was extracted from all included publications: (1) study group and year of publication; (2) segmentation methods and their outcomes (volume model, surface model); (3) segmentation technique (purely manual strategies, semiautomatic techniques or automatic segmentations based on mathematical algorithms); (4) imaging data source (CT, MRI or DSA); (5) treatment modality; (6) duration of the segmentation; (7) blood flow incorporation; (8) 3D visualization methods (screens, virtual reality, mixed reality or augmented reality).

### Statistical analysis

The descriptive statistical analyses were performed using IBM SPSS Statistics 25. Data were presented as numbers and percentages, and medians with IQR. Heterogeneity was tested by Chi-square test (significance level: *p*-value < 0.01).

## Results

**Fig. 1:**
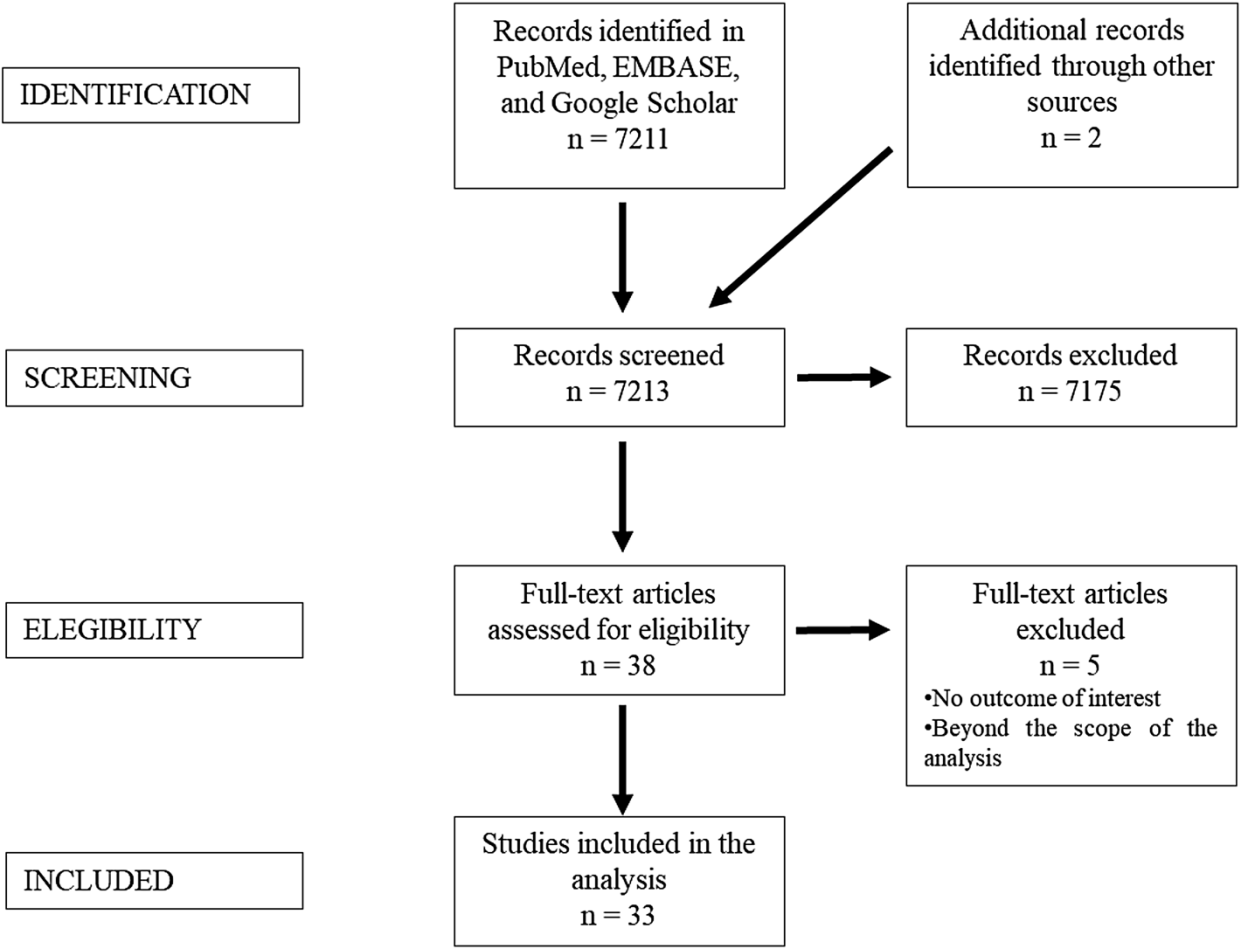
Summary of search strategy (PRISMA flow chart) for relevant studies

A PRISMA flowchart is displayed in Fig. 1. A total of 7212 publications were screened, 37 full-text articles were assessed for eligibility and 32 studies were included in this review.

### Segmentation input (Table 1)

Thirteen out of 33 studies (39%) described high-resolution magnetic resonance imaging (MRI)-based segmentation [20, 27–38]. Within this group, tridimensional time of flight-MR-Angiography (TOF-MRA) was used in 11 studies (85%): 9 studies applied a protocol without gadolinium and 2 studies with gadolinium (28, 31). Nine studies (27%) described digital subtraction angiography (DSA)-based segmentations [39–47], and 7 (21%) described native CT and CT-Angiography (CTA)-based segmentations [25, 48–53] (Table 1).

### Segmentation aim (Table 1)

Thirteen out the 33 studies (39%) aimed to render feeding arteries, the architecture of the nidus and draining veins [25, 28–30, 34, 37, 38, 43, 45, 47, 48, 50, 51]. Seven studies (21%) provided an analysis of feeders and veins, without specific focus on the nidus [18, 27, 31, 33, 49, 52, 54]. The exclusive focus on the nidus was documented in four studies (12%) [20, 21, 36, 54]. Information on the aim of the segmentation is provided in Table 1. Thirty studies (91%) implemented a segmentation strategy to achieve preoperative characterization of bAVMs; however, three studies (9%) segmented with the purpose to visualize on a navigation-linked intraoperative display [38, 55, 56].

### Manual and semiautomatic segmentation (Table 2)

**Table 2:** Overview on the segmentation strategies other that fuzzy-based methods

Technology	Machine learning	Advantages pointed out by the authors
Intersecting code model (Ref: 31, 33)	0	Reasonable approximation of the volume inside the prescription isodose line. Allows quantification of volume variations. Aids in selecting the optimal management plan. Manual to semiautomatic
Image intensity ridges (Ref: 19)	0	The approach requires a seed point for each extracted vessel, and it requires 30 to 60 min to extract all vessels from an MRA. Semiautomatic
Epipolarity geometry (Ref: 13, 32)	0	High quality and accurate localization method based on DSA examinations. Manual to semiautomatic
Principal component analysis (Ref: 41)	1—supervised	Accurate identification of the physiological location of arteries, veins and background images. Possibility to 3D reconstruction. Automatic
Integrated volume rendering (Ref: 42)	0	Precise distinction of arteries and veins, as well as identification of the nidus in spite on intracerebral hematoma. Semiautomatic
User-defined VOI (Ref: 34)	0	Integration of 2D DAS and 3D rotational angiography to process the nidus better. Automatic
Pixel neighborhood structure and intensities, and variations (Ref: 38, 43, 44)	0	Once the parameters of the analysis are fixed, the algorithm works automatically. It allows processing 2D and 3D images with high-range luminance values and noise values. It shows blood vessels precisely. Automatic
Support vector machine (Ref: 26)	1—supervised	Automatic segmentation of the seed point and reproducible and fast extraction of the bAVMs nidus and the vessels in its proximity. Automatic
Continuity propagation (Ref: 35)	0	The mean duration of the method is 64 min showing high-quality results, especially in the delineation of venous ectasias and the drainage patterns. Semiautomatic
Fractal-based computational methods (Ref: 27)	0	Reliable quantification of vascular complexity capable of nidal characterization. Manual
Region-growing algorithm (Ref: 36, 37)	0	The principal feeding arteries and draining veins connected to the nidus can be clearly identified. It enables a description of the brain vasculature in a hierarchical model, which aids to simulate the microcatheter navigation for an embolization. Automatic
Simple global thresholding (Ref: 28)	0	Elimination of noise artifacts and integration with vessel enhancement algorithms to improve the spatial analysis of bAVMs components. Manual
BrainLab (Ref: 40, 47)	0	Smartbrush function: intuitive to use. Manual
Supervised 3D V-Net with a compound loss function (Ref: 45)	1—supervised	Novel deep-learning-based method to segment a bAVM target volume on CT. It achieves higher accuracy. Automatic
3D-region-based (Ref: 39)	0	Improved 3D visualization and delineation of arteries, nidus and veins. Accurate decomposition of bAVMs structure and guidance of the embolization. Automatic
Convolutional neural network (Ref: 29)	1—supervised	Accurate delineation of bAVM components, as well as brain parenchyma, CSF and embolized vessels across an anatomically variable validation set. Automatic
Horos Software (Ref: 30)	0	Useful for preoperative 3D reconstruction allowing accurate delineation of bAVM major components Manual

Seven studies (21%) described manual bAVM segmentation [21, 34, 35, 38, 39, 48, 55], and 6 studies (18%) described semiautomatic algorithms [27, 40, 43, 54] In this subgroup, 3 studies (23%) aimed for delineation of all the bAVM components [34, 38, 48], while the other 10 studies (77%) focused on the segmentation on a single component of the bAVM or on the volume of the lesion. Four semiautomatic segmentation studies documented a median duration of 25 (IQR 73) minutes [27, 43, 51, 54].

### Automatic segmentation (Table 2)

Twenty studies (61%) used an automatic mathematical algorithm to segment bAVMs [20, 25, 28–33, 36, 37, 42, 44–47, 49, 51–53, 56]. Eight of these studies (40%) aimed to segment all three bAVM components [25, 28–30, 37, 45, 47, 51]. Median segmentation time was 10 min (IQR 33), described in 6 out of the 20 studies. Eight automatic segmentation studies (40%) performed segmentation by an unsupervised fuzzy-based method, with a median processing time of 10 min (IQR 33) [20, 28–32, 36]. Only 1 research group further provided a hemodynamic characterization of the segmented bAVM components. Hemodynamics were provided by integrating temporal blood flow information of the vessels in proximity of the nidus [32, 33].

Other groups included in this cohort performed an automatic image segmentation based on supervised methods [37, 49, 53]. These strategies included supervised principal component analysis [49], supervised 3D V-Net with a compound loss function [53] and supervised convolutional neural network [37].

The most utilized imaging input in the automatic segmentation subgroup was MRI (10/20, 50%).

### Segmentation variability

A Chi-square test of homogeneity was performed to examine the methodological variability of the collected studies. The homogeneity between the segmentation strategies and the reported outcomes, namely segmentation of all bAVM components, segmentation of the nidus only or other outcomes (e.g., volume), was not significant, with a Chi-squared = 0.13 (*p*-value 0.99).

### 3D visualization

Four studies (12%) described an integration of their segmentation strategies with mixed reality for intraoperative guidance under the microscope [37, 47, 55, 56]. While integration with AR was positively endorsed, the future necessity to integrate hemodynamic considerations to the AR rendering to provide more useful and precise intraoperative information was also suggested [18]. None of the included studies aimed to a rendering of the segmentation outcomes with VR.

### Clinical value and blood flow analysis

In 19 studies (58%), the final goal of segmentation was an accurate and more precise planning and intraoperative management of bAVM [25, 27–33, 35, 38, 43, 45, 48, 50–52, 54–56]. Eleven studies (33%) aimed to a better delineation of bAVM components to achieve a more effective and safer radiosurgical treatment [20, 21, 34, 36, 37, 39–42, 49, 53]. Three (9%) of the included studies set embolization as the final goal of segmentation and better angioarchitecture visualization [44, 46, 47].Only one research group aimed and achieved the inclusion of hemodynamic considerations to 3D bAVM visualization. They could provide temporal information on blood flow of the vessels in the close proximity of the nidus by calculating a time-to-peak parameter map and registering it to TOF-MRA sequences [32, 33].

## Discussion

The present study represents a systematic analysis on segmentation techniques of bAVMs for 3D visualization and accurate angioarchitectural study. This systematic review focusses on 3D bAVM segmentation and visualization strategies, which have been published from 1997 to 2022 on the characterization of the angioarchitecture of bAVMs. Although most studies use MRI as input and a tendency was shown in the direction of automatic machine learning trained algorithms, this review shows how significantly variable the possible methods of segmenting a bAVM are, and with this the absence of a gold standard.

Due to methodological variety of manual, semiautomatic and automatic segmentation techniques and their outcomes, the results should be interpreted with caution. Intrinsic biases of included publications cannot be ruled out. The average segmentation duration was evaluated using very limited data from only a few studies. Because of the great variability of the data collected in the present systematic review, a descriptive statistical analysis was performed. Given the technical and preclinical nature of most of the included studies, very few of them documented sensitivity and specificity of the segmentation method. Therefore, the scarcity of statistical data made the performance of a pooled analysis impossible.

This review describes a tendency over time to base bAVM segmentation on algorithms trained with machine learning, especially deep learning, because manual segmentations are prone to human error and interindividual assessment and are labor intensive [28]. Algorithms trained with machine learning stem from two principles: supervised and unsupervised learning. Supervised learning algorithms, such as support vector machine, have the disadvantage of relying on manual segmentations. In addition, the variability of scans should be large, because algorithms trained on scans from 1 center are rarely easily extrapolated. Unsupervised learning does not require initial information, and algorithms based on this technology are generally fast. In the present analysis, fully automated segmentation via unsupervised learning has good results using fuzzy-based methods. This approach is based on the computation of vesselness filter and maximum parameter images on MRI-TOF sequences, providing a highly precise delineation of large as well as fine vessels [32, 36].

The deep-learning algorithm developed by Forkert et al. describes the extra advantage of producing surface models of the vascular system, which can be not only visualized but also manipulated in 3D [28, 32]. In the settings of 3D segmentation, operability of surface models differentiates them from outcomes of computational heavier volumetric models, that in general are more detailed and look sharper than surface models but do not allow any interaction and are mostly too heavy to visualize in a head-up display without streaming [57]. In view of extensive integration of medical imaging with mixed reality or virtual reality in the future, the use of surface models versus volume models as a possible output of algorithms in highly complex 3D lesions as bAVMs should be further investigated.

In a medical world moving toward personalized medicine, segmentation strategies and 3D imaging visualization techniques are increasingly gaining popularity. Pre- and intraoperative delineation of complex anatomical entities like bAVMs with these technologies provides important clinical advances [58]. First is a more precise understanding of bAVM angioarchitecture and anatomical relationships with the surrounding structures [37, 38]. Therefore, a more accurate and individualized therapy planning and the possibility to achieve more efficient patient management and potentially better clinical outcomes [56]. Only one research group incorporated flow in 3D bAVM segmentation. However, flow is extremely important in assessing brain AVM (re-)rupture risk analysis [59]. Transcranial Doppler (TD), DSA and quantitative MR-Angiography (QMRA) [60, 61] have been described as useful techniques to analyze flow in bAVM [22, 62–64]. The integration of these techniques in 3D bAVM segmentation should be a future goal.

## Conclusion

A golden standard for 3D visualization of bAVMs does not exist. This review describes a tendency over time to base segmentation on algorithms trained with machine learning. Unsupervised fuzzy-based algorithms thereby stand out as potential preferred strategy. Continued efforts will be necessary to further improve algorithms, integrate complete hemodynamic assessment and find new innovative tools for tridimensional visualization.
